# Productivity and genetic stability of a novel baculovirus vector for multigene expression from independent transgene loci

**DOI:** 10.1016/j.omta.2026.201668

**Published:** 2026-01-09

**Authors:** Linda A. de Jong, Mels Schrama, Jelmer Willems, Adriaan H. de Wilde, Corinne Geertsema, Marleen Henkens, Dirk E. Martens, Barbara P. Sanders, Monique M. van Oers, Gorben P. Pijlman

**Affiliations:** 1 Laboratory of Virology, Wageningen University & Research, Droevendaalsesteeg 1, 6708 PB Wageningen, the Netherlands; 2 Department of Bioprocess Engineering, Wageningen University & Research, Droevendaalsesteeg 1, 6708 PB Wageningen, the Netherlands; 3 VectorY Therapeutics, Science Park 408, 1098 XH Amsterdam, the Netherlands

**Keywords:** genome editing, bacmid, baculovirus expression vector system, illumina next generation sequencing, SARS-CoV-2, adeno-associated virus, AAV

## Abstract

The baculovirus expression vector system is an established platform for large-scale production of (glyco)proteins, subunit vaccines, virus-like particles, and recombinant adeno-associated virus (rAAV) vectors. We engineered a novel bacmid vector (BAC6) to improve genetic stability by deletion of the non-homologous repeat (hr) origin of DNA replication (ori) and to preserve product integrity and recovery through deletion of chitinase and cathepsin. Tn7-based transposition in *E. coli* and homologous recombination in insect cells were combined in BAC6 to drive expression from the odv-e56 and polyhedrin loci, respectively. Virus growth kinetics of BAC6 were similar to the parental bacmid, and genetic stability was investigated for at least eight serial passages at high multiplicity of infection. Next-generation sequencing was used to identify mutations, deletions, and defective interfering particle (DIP) formation, which became apparent only in later passages. With BAC6, the enrichment of DIPs originating from the non-hr ori was prevented. BAC6 versatility was demonstrated by high-yield (12 mg/L) severe acute respiratory syndrome coronavirus 2 (SARS-CoV-2) spike production in suspension Sf9 insect cells. Finally, rAAV production with BAC6, simultaneously employing both transgene insertion sites, resulted in yields of 5.8e10 AAV capsids/mL and 2.3e10 genome copies/mL. BAC6 provides insertion of multiple transgenes at two different loci and is non-inferior to commercial baculovirus expression vectors with regard to genetic stability and productivity.

## Graphical abstract

**Figure:**
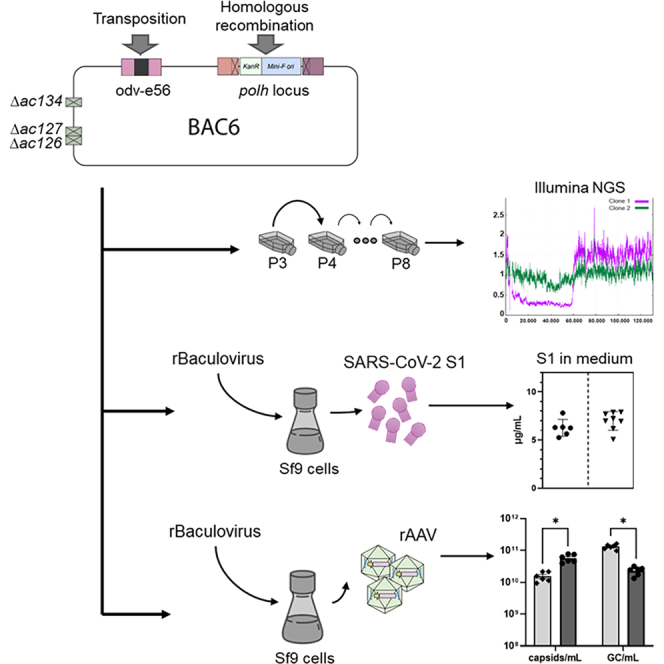

## Teaser

De Jong and colleagues present a novel baculovirus vector for multigene expression from independent transgene loci. They study baculovirus genetic stability with next-generation sequencing and demonstrate the productivity of the SARS-CoV-2 spike S1 domain and recombinant adeno-associated virus vectors in insect cell suspension culture.

## Introduction

The baculovirus expression vector system (BEVS) is an attractive platform for the production of recombinant proteins. The availability of strong promoters, compatibility with suspension insect cell culture, and extensive knowledge of its biology mean that baculovirus expression technology has been widely established and applied, for example, for the production of a subunit vaccine against classical swine fever virus,^1^ virus-like-particle vaccines against porcine circovirus^2^^,^^3^ and human papillomaviruses,^4^^,^^5^ adeno-associated virus (AAV) vectors for gene therapy,^6^^,^^7^ and severe acute respiratory syndrome coronavirus 2 (SARS-CoV-2) spike S1 domain subunits for display on nanoparticle vaccines.^8^^,^^9^ Through the introduction of a bacterial origin of replication (ori) and an antibiotic resistance marker, baculovirus DNA can be propagated as a single-copy “bacmid” in *E. coli*^10^ allowing not only for transgene insertions but also for genome modifications through methods such as lambda red recombineering.^11^^,^^12^^,^^13^^,^^14^ Most of the BEVS applications make use of the *Autographa californica* multiple nucleopolyhedrovirus (AcMNPV), though *Bombyx mori* nucleopolyhedrovirus is also frequently used for foreign gene expression,^15^ and bacmids for *Spodoptera exigua* multiple nucleopolyhedrovirus,^14^ and *Helicoverpa armigera* single nucleopolyhedrovirus^16^ also exist. Transgene sequences are often inserted at the polyhedrin (*polh*) locus, originally through homologous recombination by co-transfection of viral genomic DNA with a transfer plasmid in insect cells,^17^ a method later optimized by linearizing the baculovirus genome before transfection.^18^ Alternatively, transgenes can be introduced into bacmid DNA through Tn7-mediated transposition in *E. coli*.^10^ The transposition-compatible AcMNPV bacmid bMON14272 has been commercialized as the “Bac-to-Bac” system (Invitrogen), whereas homologous recombination with linearized baculovirus DNA is the technique used in optimized classical systems such as “*flash*BAC” (Oxford Expression Technologies), “BacMagic” (Novagen), “BacPAK6” (Takara), “BestBac” (Expression Systems), ”Bac-N-Blue” (Invitrogen), and BaculoGold (BD BioSciences).

Despite the ever-growing number of baculovirus applications in the field of biotechnology, there are known problems associated with the BEVS. Most notably, the instability of the baculovirus genome in cell culture could result in loss of the transgene from the genome and subsequent reduction of recombinant protein production levels over viral passages.^19^^,^^20^^,^^21^^,^^22^ Wild-type AcMNPV produces two types of progeny virus: budded virus (BV) for spreading from cell to cell in the insect or insect cell culture, and occlusion-derived virus (ODV), which is important for oral infectivity of insects.^23^ Over half of the coding sequences in the baculovirus genome are thought to be non-essential for the production of progeny BV in cell culture.^24^ The presence of sequences redundant for BV production, and hence for cell culture infection, may contribute to the generation (and selection) of viral deletion mutants, which often arise upon sequential passaging of virus stocks. In a phenomenon known as the “passage effect” or the “Von Magnus effect,” partial viral genomes known as defective interfering particles (DIPs) are generated over passage of viruses from several different families, first reported for influenza virus.^25^ Baculovirus-derived DIPs interfere with the infection kinetics of virus particles with intact genomes and cause significant reductions in transgene expression.^26^ Studying the replication dynamics between the helper and defective baculoviruses demonstrated that DIP-contaminated stocks titers show an irregularly oscillating pattern upon extended serial passaging.^27^ There is no definitive cause determined for baculovirus DIP formation, although some regions within the genome are known or suspected to contribute to the phenomenon. Previous studies have shown that deletion of specific regions can enhance the stability of the AcMNPV genome and delay DIP formation, such as the egt (*ac15*) and da26 (*ac16*) loci,^21^^,^^28^ the *fp25k* gene (*ac61*),^29^^,^^30^ and the non-homologous repeat ori (non-hr *ori*) within the *p94* gene (*ac134*).^14^ Aside from improving the genomic stability, the deletion of particular open reading frames (ORFs) has also been shown to significantly increase transgene expression levels.^31^^,^^32^ Furthermore, some deletions can benefit the integrity of the produced proteins. For example, deletion of the chitinase (chiA; *ac126*) and cathepsin (v-cath; *ac127*) genes was shown to improve product secretion and integrity.^33^

In this study we aimed to develop a novel, genetically stable baculovirus vector (bacmid) that is compatible with the incorporation of transgenes at two different loci. Here we present a novel bacmid, named BAC6, in which the two most commonly used techniques to generate recombinant baculoviruses, Tn7-based transposition and homologous recombination in insect cells, are integrated into a single bacmid, offering multiple transgene insertions at different loci. BEVS allowing the insertion of multiple transgenes have been around, for example, in the “MultiBac” system, where Cre-loxP recombination and Tn7 transposition were combined at two different loci in the same bacmid. The main downside of this system is the retainment of the mini-F origin next to the transgene sequence, which was found to be a source of instability.^34^ BAC6 overcomes these challenges during the homologous recombination step where the mini-F replicon is removed entirely and replaced with a transgene sequence. BAC6 was generated through lambda red recombineering. At the odv-e56 locus (*ac148*), an attTn7 site was introduced to allow for transgene introduction through Tn7-mediated transposition in *E. coli*.^10^^,^^35^ Next, partial deletions were introduced in ORFs 603 and 1629. The partial deletion in ORF1629 renders the bacmid replication-incompetent unless recombination with the transfer plasmid occurs, thereby preventing non-recombinant parental virus from contaminating. Additionally, insertion of a transgene through homologous recombination in insect cells at the *polh* locus will in this way also effectively remove the mini-F replicon sequences that are known to be genetically unstable upon passaging.^34^

To further improve the genetic stability during passaging, the non-hr ori in p94 (*ac134*) was deleted.^14^ Additionally, the chiA (*ac126*) and v-cath (*ac127*) genes were removed to safeguard the integrity of expressed recombinant proteins.^33^^,^^36^ BAC6-derived recombinant baculoviruses were investigated for genomic stability and ability to express mCherry during a high multiplicity of infection (MOI) passaging study, as well as used to test the production of two additional recombinant products: the S1 domain of the SARS-CoV-2 spike protein and recombinant (r)AAV. The detection and identification of DIPs within virus populations was conducted by a new next-generation sequencing (NGS) approach.

## Results

### BAC6 was successfully generated and validated and can be used to generate recombinant virus

**Figure 1:**
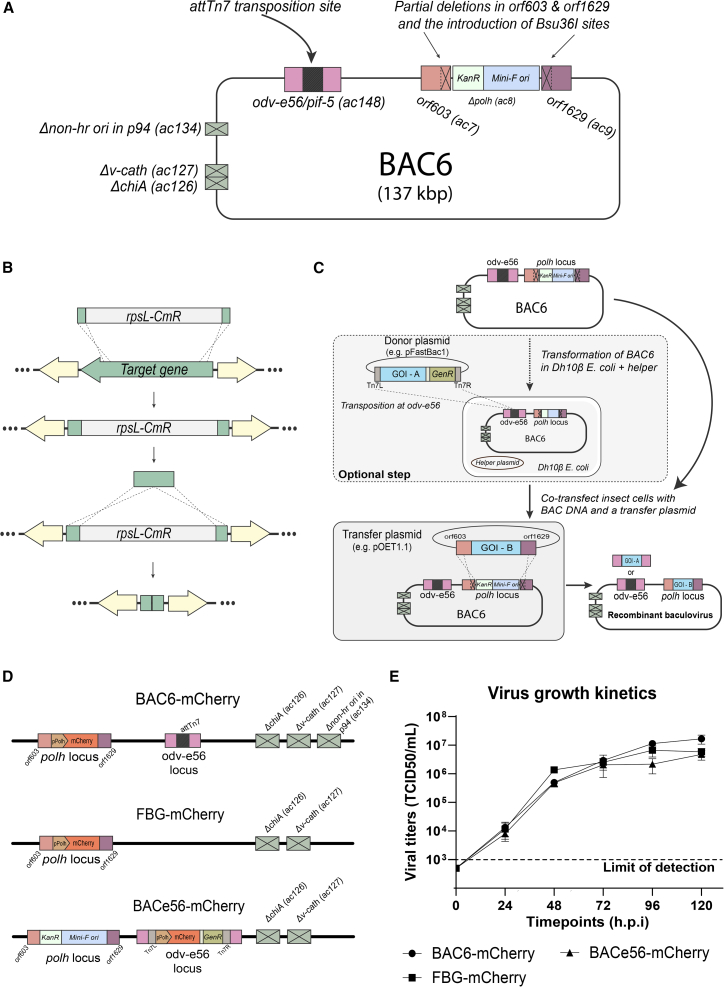
Construction of the novel recombinant bacmid BAC6 (A) schematic overview of bacmid BAC6 that was developed and tested in this study. (B) The mutations were made through lambda red recombination in *E. coli*. An antibiotic resistance-sensitivity cassette, rpsL-CmR, with homology flanking to the region to-be-deleted was electroporated into *E. coli* harboring a lambda red operon and the bacmid. Positive selection on chloramphenicol was used to screen recombinants. To remove the cassette, the electroporation was repeated with a fragment of the flanking regions. Negative selection was performed on streptomycin. (C) The incorporation of transgenes in the novel bacmid is performed through two methods: transposition at odv-e56 in *E. coli* and/or homologous recombination at the polyhedrin (polh) locus on insect cells. The former is an optional step, whereas the latter is mandatory due to the required restoration of ORF1629 in order to generate a replication competent virus. (D) To study the growth kinetics, three different recombinant viruses were generated. For BAC6-mCherry and FBG-mCherry, the transgene cassette of mCherry driven by a polh promoter was introduced through homologous recombination at the polh locus. For BACe56-mCherry, this was done through transposition at the odv-e56 locus. In BAC6, this attTn7 transposition site is also present at the odv-e56 locus, but no transgene was introduced here. In all three viruses, the v-cath and chiA genes have been deleted. In BAC6-mCherry there is an additional deletion of the non-hr ori in p94. (B) Side-by-side growth kinetics of BAC6-mCherry, FBG-mCherry, and BACe56-mCherry. Three biological replicates were included during the assay. Samples were taken at 0, 24, 48, 72, 96 and 120 hpi. The data show the mean ± SEM (standard error of the mean) of triplicates. ORF, open reading frame; polh, polyhedrin; v-cath, cathepsin; chi-A, chitinase; non-hr ori, non-homologous region origin of replication).

Through subsequent lambda red recombineering in *E. coli*, the novel bacmid BAC6 containing two transgene loci and multiple gene deletions was generated successfully (Figure 1A). Each recombination intermediate (Table 1) was validated by PCR and sanger sequencing (data not shown), and the sequence of the final intermediate, BAC6, was validated with Illumina NGS. Transgenes can be introduced into the bacmid either through an optional transposition step at the odv-e56 locus or through homologous recombination at the *polh* locus, which is mandatory (Figure 1C). To further characterize BAC6 and investigate whether the introduced deletions and modifications affect the replication kinetics of the virus, growth curves were generated of BAC6 in comparison to the two reference viruses harboring the same transgene. These include *flash*BAC Gold (FBG; Oxford Expression Technologies) and BACe56^35^ (Figure 1D). All three viruses contain an mCherry transgene under the *polh* promoter, introduced either through transposition in *E. coli* (BACe56) or through homologous recombination in insect cells (BAC6, FBG). Time point samples were taken from the supernatant from 0 to 120 hour post infection (hpi) at 24 h intervals, and titers were scored based on green fluorescent protein (GFP) expression (from the host cell genome). The growth curves show comparable growth kinetics between the three viruses included in the experiment at any time point (Figure 1E), thus proving that the modifications and deletions in BAC6 did not affect its replication kinetics.

**Table 1:** Overview of the sequential mutations or deletions that were introduced into bacmid BAC6 and the intermediates that were generated in this study

orf → intermediate ↓	orf603 (ac7)	*polh* (ac8)	orf1629 (ac9)	chiA (ac126) & v-cath (ac127)	Non-hr ori in p94 (ac134)	Odv-e56 (ac148)	Total bp deleted
bMON14272 (starting material)		attTn7 and Mini-F					–

**Genomic deletions**							

BAC1		attTn7 and Mini-F	Δ673 bp				Δ673 bp
BAC2	Δ512 bp	attTn7 and Mini-F	Δ673 bp				Δ1185 bp
BAC3	Δ512 bp	attTn7 and Mini-F	Δ673 bp	Δ1930 bp			Δ3115 bp
BAC4	Δ512 bp	attTn7 and Mini-F	Δ673 bp	Δ1930 bp	Δ2055 bp		Δ5170 bp

**AttTn7 relocation from polh locus to odv-e56**							

BAC5	Δ512 bp	Δ and Mini-F	Δ673 bp	Δ1930 bp	Δ2055 bp		Δ5546 bp
BAC6	Δ512 bp	Δ and Mini-F	Δ673 bp	Δ1930 bp	Δ2055 bp	attTn7	Δ5170 bp

The bMON14272 bacmid described in ref ^10^ was selected as the starting material. The starting material is the AcMNPV genome (strain C6), where at the polyhedrin (polh) locus the coding sequence is replaced with a bacterial origin of replication (Mini-F replicon), a kanamycin resistance gene, a lacZ gene and an attTn7 docking site. (ORF, open reading frame; v-cath, cathepsin; chi-A, chitinase; non-hr ori, non-homologous region origin of replication).

### Analysis of transgene retainment during serial undiluted passaging

**Figure 2:**
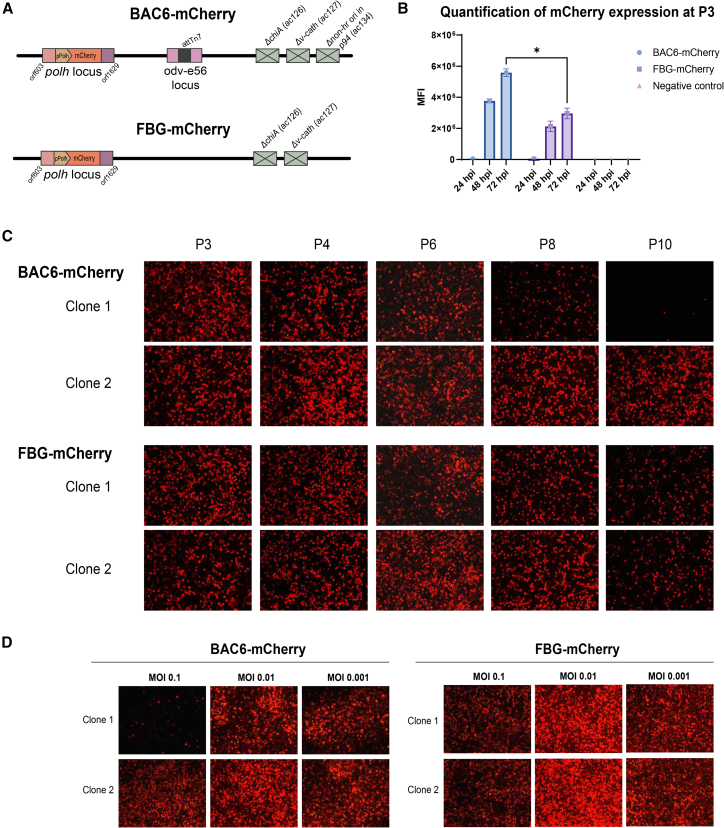
Transgene expression and high MOI passaging study on adherent Sf9 cells (A) The viruses included in the study are BAC6 and flashBAC Gold (FBG), both of which express a polyhedrin (polh) promoter-driven mCherry reporter gene from the polh locus. (B) Flow cytometry quantification of mCherry produced with BAC6-mCherry and FBG-mCherry. Expression was measured in mean fluorescence intensity (MFI) at 24, 48, and 72 hpi. Significance was determined by an unpaired two-sample *t* test in Graphpad Prism Version 10.4.2. ∗: *p* < 0.05. The data show the mean ± SEM of experimental triplicates. (C) Imaging of the infected Sf9 cells through the rhodamine channel during the passaging experiment at passages (P) 3, 4, 6, 8, and 10. (D) Low MOI infections on monolayers of Sf9 cells with the final passage stocks. Three MOIs were tested: 0.1, 0.01, and 0.001, based on the GFP readout of the EPDA titers on Sf9-ET cells. Imaging was done 5 days post infection.

To investigate the genomic stability of the novel bacmid, a passaging study was conducted in adherent layers of Sf9 cells at high MOI. BAC6-mCherry and FBG-mCherry (Figure 2A) viruses were generated by co-transfecting bacmid DNA with a transfer plasmid to insert an mCherry reporter transgene under a *polh* promoter at the *polh* locus through homologous recombination. Prior to starting the passaging study, the mCherry protein expression levels were compared between the viruses using a flow cytometric method. The P3 virus stocks were used to infect suspension cultures of Sf9 cells at an MOI of 5. The mCherry transgene expression was analyzed with flow cytometry (Figure 2B). At 24 and 48 hpi, the two viruses showed comparable levels of transgene production. At 72 hpi, the mean fluorescence intensity (MFI) of mCherry measured with BAC6-mCherry was significantly higher compared to the FBG-mCherry virus, as determined with an unpaired two-sample *t* test (Graphpad Prism, version 10.4.2). Next, two clones per virus were serially passaged until P10 was reached. Visualization with the fluorescence microscope was done at every passage, and the images at P3, P4, P6, P8, and P10 are depicted in Figure 2C. All viruses retained high mCherry production for at least 6 serial passages, as scored by visual inspection of the microscopy images. In clone 1 of BAC6-mCherry, a reduction in fluorescent signal was observed starting at P8, further decreasing strongly until P10, indicating loss of transgene from the prevalent baculoviral genome. Decreased fluorescence was also detected in both clones of FBG-mCherry at P10. The decrease in fluorescence can likely either be attributed to a loss of the mCherry transgene sequence or due to the formation of DIPs within the stock.

Next, low-MOI infections were conducted to evaluate the potential recovery of infectious viruses from DIP-contaminated P10 stocks. Using a low MOI dilutes out DIPs, allowing intact viruses to replicate efficiently and restore a functional virus population. Three MOIs were tested: 0.1, 0.01, and 0.001 and the degree of infection was assessed by visual expression of mCherry under the fluorescence microscope (Figure 2D). Five days post infection and at all three MOIs, infection of the cell culture was observed with both clones of FBG-mCherry and with the BAC6-mCherry clone 2. At the same time point, when infecting at an MOI of 0.1, complete infection of the culture was not observed for BAC6-mCherry clone 1. Lowering the MOI further to 0.01 and 0.001 does restore the full infectivity of this virus stock. If DIPs have accumulated in the virus stock, it is to be expected that at an MOI of 0.1 these particles cause interference and competition with the intact virus. The fact that this problem is overcome at low MOI infections provides further affirmation that the observations made for BAC6-mCherry clone 1 are due to DIP formation.

### Titration assays elucidate DIP accumulation in one BAC6 clone and loss of the transgene sequence in both FBG clones at later passages

Next, we sought to investigate the titers that were used during passaging and test if we could detect transgene loss and/or DIP formation to complement the previous microscopy observations. To test this, we analyzed the viruses using three complementary titration methods. First, GFP-based TCID50 was used to measure infectious titer. Second, mCherry readout was used to measure the titer of infectious virus harboring the mCherry transgene. Third, as a measure of infectious virus particles (ivp), GP64 expression was detected on the surface of infected cells.

**Figure 3:**
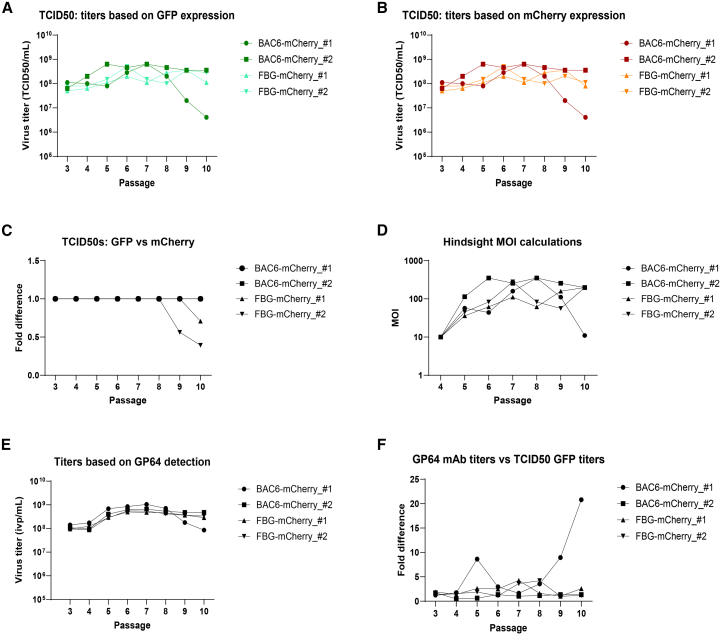
Titers of the virus stocks included in the high MOI passaging study from P3 until P10 The samples were titrated with EPDA (TCID_50_/mL) on Sf9-ET cells, and the readout was based on (A) GFP or (B) mCherry. (C) Comparing the two readouts indicates whether all the infectious virus in the stock expresses the mCherry transgene. (D) The EPDA titers based on GFP expression were used to calculate the MOIs that were used during the previous passage. (E) A second titration method based on GP64 detection on the surface of infected cells using a monoclonal antibody (mAb) was also performed, giving titers in infectious virus particles per mL (ivp/mL). (F) Comparing the GP64 mAb titers to the EPDA titers (GFP readout) shows fluctuations, indicative of virus populations that no longer produce infectious virus but are able to enter a cell and express GP64.

The harvests of the genetic stability samples after each passage were titrated by endpoint dilution assay (EPDA) on Sf9-ET cells, and the first readouts were based on GFP expression (Figure 3A), as well as on mCherry expression (Figure 3B). Comparing the titers based on GFP (triggered in the cells by the presence of a replicating virus) to those based on mCherry (encoded in the virus) gives insight into how well the mCherry transgene was retained over passages (Figure 3C). The clones of both viruses had an identical titer for both GFP and mCherry readouts until P8. At passages 9 and 10, the mCherry titer readout started to decline compared to the enhanced GFP (eGFP) readout for the FBG-mCherry viruses, thus indicating that a loss of transgene was likely occurring from P9 onwards. In contrast, the mCherry and GFP readouts for the BAC6-mCherry clones remained the same until the final passage, indicating good transgene retention.

The GFP titer was also used to calculate the MOI over passages (Figure 3D) in hindsight, since the passaging was performed at fixed volumes. The hindsight calculations confirmed the MOIs during passaging were indeed varying from high to very high (10<MOI<400).

The second titration method was performed through the detection of GP64 on the surface of infected cells using a monoclonal antibody. Viral titers were then measured with the flow cytometer in ivp/mL (Figure 3E). The GP64 mAb titers were compared to the GFP readout in the EPDA (Figure 3F), showing for all samples similar titers in both assays, except for sample BAC6-mCherry clone 1. This sample shows at P5 a peak indicating a 5-fold higher GP64 mAb titer compared to the EPDA. This peak disappears at P6 but returns at P9 and P10, increasing to an 8- and 20-fold difference, respectively. This comparison between the two titration methods gives insight into the infectious replication-competent virus particle population (EPDA: TCID_50_ units/mL) versus the infectious total virus particle population (GP64 mAb titration). The difference between the two is indicative of a defective population of viruses in the stock that no longer have the ability to generate new BV but are able to enter the cell and express GP64 from the early gp64 promotor.^37^ The findings from the titration assays are in line with the earlier observations during the passaging imaging and the low MOI infections (Figures 2C and 2D).

### Illumina NGS reveals the formation of different virus populations in all viruses that were serially passaged

**Figure 4:**
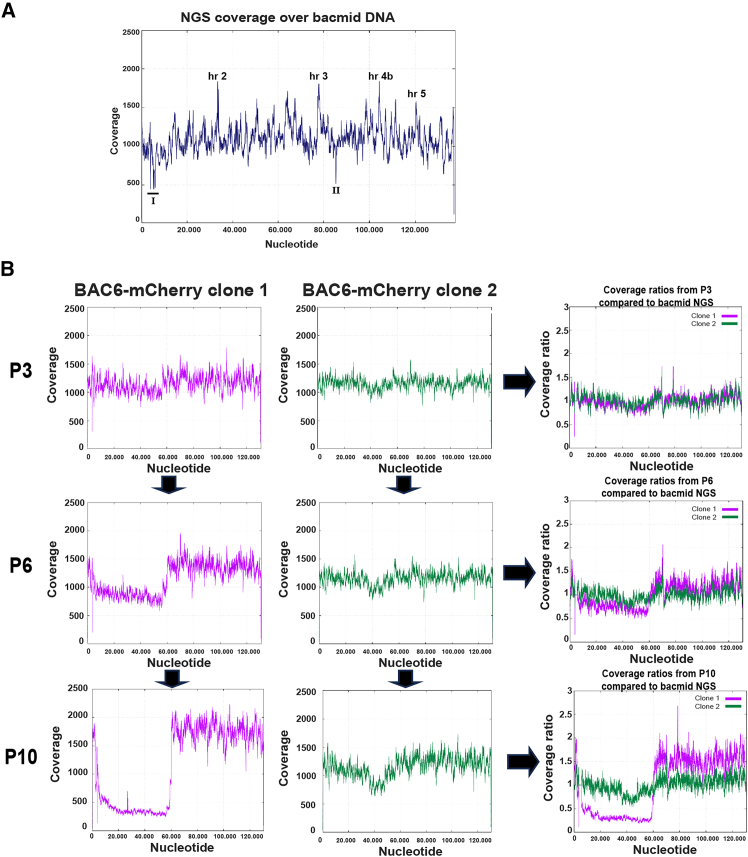
Illumina NGS analysis on the BAC6 bacmid DNA and the viral DNA after high MOI passaging (A) Coverage over each nucleotide of the BAC6 bacmid DNA. Indicated in the graph are the peaks corresponding to homologous repeat (hr) regions in the genome. The location of the kanamycin gene and mini-F replicon are indicated with I. A region with a drop in coverage corresponding to ORF ac91 is indicated with II. (B) Illumina Next Generation Sequencing on P3, P6, and P10 viral DNA isolated from infected cells during the high MOI genetic stability passaging study. The normalized coverage of the NGS data mapped to a reference sequence is shown for the samples at P3, P6, and P10. Ratio plots were made by dividing the coverage of the samples over each nucleotide at P3, P6, and P10 by the coverage of the bacmid NGS read coverage. The viral DNA contains an mCherry transgene at the polyhedrin locus, which is not present in the bacmid NGS. To negate this in the plot, the coverage ratios in this region (3861–5525 nt) were adjusted to 1. Read coverage was normalized between the different samples to correct for differing sequencing depths.

To confirm the results seen during the titration assays and confirm the formation of DIPs is occurring, an NGS approach was employed. In addition, if DIPs are accumulating, with NGS we aim to elucidate which regions are responsible. Genomic analyses of BAC6 viral DNA over passages 3, 6, and 10 were performed by Illumina short-read sequencing. In addition, the bacmid DNA isolated from *E. coli* was sequenced to generate a reference sequence that can be used as a control to compare the BAC6 genomic sequences to after the serial passaging shown in Figure 2C. This also helps to determine whether observations made in the NGS plots of the viral DNA are due to actual differences occurring during passages or if they are due to difficulties in the sequencing over certain regions of the genome. The normalized read coverage over the bacmid genome was plotted in Figure 4A. The read distribution over the genome shows some notable peaks that we investigate in more detail. The peaks correspond to regions that are covered by more reads compared to the rest of the genome. Specifically, the peaks around 33.000 , 77.000, 104.000, and 120.000 nucleotides (nt) correspond to *hr* regions *hr 2*, *hr 3*, *hr 4b*, and *hr 5*, respectively. Since these regions consist of highly similar and AT-rich repeat regions,^38^ they can cause a bias when sequencing with Illumina due to biased fragmentation, PCR amplification, and cluster formation. Another possibility is that, since the reads over the repeat regions are identical or highly similar and they were only allowed to map once, the Segemehl tool used for mapping forced them into the same position where they fit best, thereby causing the peaks on the graph.

Apart from the hr regions, decreased coverage was observed at two notable locations. The first is from around 3800 to 6700 nt, which is the region where the kanamycin resistance gene is located, as well as the first ∼1500 nt of the Mini-F replicon (indicated with I in Figure 4A). The second drop in coverage is around 85.200 nt, which corresponds to ORF *ac91* (indicated with II in Figure 4A). Possible reasons for the decreased coverages here could be due to a sequence complexity bias, or these regions contain a short repeat that is also present elsewhere in the genome, thereby causing a lower coverage in the graph.

Next, to obtain an in-depth view of the virus populations within the stocks after serial undiluted passaging, we investigated changes in genome coverage over baculovirus passages. Viral DNA from BAC6-mCherry (clone 1 and clone 2) isolated from infected cells at passages 3, 6, and 10 was sent for Illumina NGS (Macrogen Europe). After mapping the reads to the reference sequence, the coverage over each nucleotide was extracted and plotted for both clones (Figure 4B). The coverages at passages 3, 6, and 10 were also divided by the average coverage over each nucleotide in the bacmid NGS, and the resulting ratios were plotted on the right (Figure 4B). In BAC6-mCherry clone 1, there is a relatively higher coverage over the part of the genome from ∼60.000 nt onward. This difference is further amplified in the plots for P6 and P10, indicative of a growing population of deletion mutants within the virus stock. Since this population appears to lack approximately ∼44% of the genome, including multiple genes that are essential for BV production, these can be considered as DIPs that were generated during the high MOI passaging. DIP formation would also explain the previous observations made during the monitoring during passaging (Figure 2C), the low MOI infections (Figure 2D) and the discrepancies between the EPDA and GP64 mAb titrations (Figure 3F). The same is not observed for BAC6-mCherry clone 2, since for this clone at P3 no abnormalities stand out. Instead, at passages 6 and 10, a decrease in coverage over the genome from around 35.800 to 47.800 nt is observed, likely indicating a growing subpopulation with a deletion of this region in the genome. This deletion spans from approximately ORF *ac43* until *ac58*. Within this region of the genome fall the ORFs for *ac53*, *ac53a* (late expression factor 10 [LEF-10]), and *ac54* (VP1054), which are essential for replication and/or nucleocapsid assembly. Therefore, this is likely also a population of DIPs being formed, indicating that both virus clones show DIP formation, although the deletions are different. NGS sequencing unveils that both clones undergo DIP formation and that the identities of these DIPs are different. Nonetheless, these results are in line with titration experiments/imaging, bringing further confirmation of observed results being attributable to DIPs.

**Figure 5:**
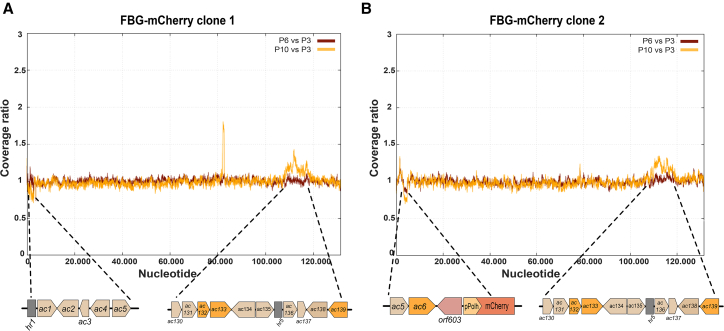
FBG-mCherry NGS: Illumina Next Generation Sequencing on P3, P6, and P10 viral DNA isolated from infected cells during the high MOI genetic stability passaging study The coverage over each nucleotide at passages 6 and 10 was divided by the coverage at P3, shown for (A) clone 1 and (B) clone 2. The regions with strongly differing coverages, either increased or decreased, compared to P3 are indicated. The ORFs indicated in orange are essential for budded virus generation or virus replication.

Next, the genetic stability of the commercial control was investigated at P3, P6, and P10. The ratio plots in Figure 5 show the read coverages over the genome at passages 6 and 10 divided by the read coverages at P3 for each respective clone. By plotting the data like this, the differences that have occurred since P3 during passaging are highlighted.

In FBG-mCherry Clone 1 (Figure 5A) the ratio plot shows a nearly consistent ratio of 1 at P6; however, at P10 there are some notable differences. We found a decreased coverage at the start of the genome, starting in hr 1 (∼140 nt) and encompassing ORFs *ac1* until *ac5* (∼3000 nt), indicating a virus population with a deletion of this region. Both hr 1 and *ac1* until *ac5* are non-essential for virus replication or BV generation. In clone 1 around 82.000 nt there is a nearly 700 nt region with increased coverage in the ratio of P10 vs. P3. This is within ORF *ac95*, which encodes for the helicase. This peak is not observed in P3 or P6 material of clone 1 and not in any of the coverages in clone 2 either and was not further investigated. Additionally, there is an increase in coverage from ORF *ac130* (∼108.000 nt) until halfway through *ac139* (∼119.500 nt). This increase in coverage is detected in the same region in clone 2 as well and could be indicative of possible DIPs that formed within the virus stocks during passaging.

Finally, in clone 2 a loss of (part of) the transgene sequence is detected from ∼3000 until 4700 nt (Figure 5B). This is observed in P6 and more amplified in P10 and is in line with the observations made during passaging (Figure 2C) and titrations (Figures 3A–3C), although in those datasets it was not picked up until P8/P9. Within this region also falls lef-2 (*ac6*), which is essential for viral DNA replication and very late gene expression.^39^

The genetic stability study results demonstrated that with respect to genetic stability and transgene stability, the BAC6 bacmid is non-inferior compared to the commercial baculovirus expression vector.

### SARS-CoV-2 spike S1 domain production by BAC6

**Figure 6:**
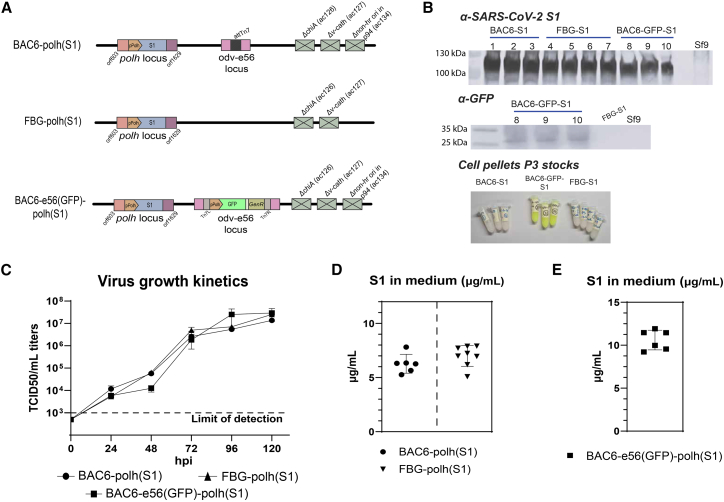
SARS-CoV-2 S1 domain production in insect cells (A) Overview of the three viruses included in the study. All contain an expression cassette with a polyhedrin promoter and the SARS-CoV-2 spike S1 domain introduced at the polyhedrin locus through homologous recombination. The BAC6-e56(GFP)-polh(S1) virus additionally expresses an eGFP driven by a polyhedrin promoter from the odv-e56 locus. Three or four plaque-purified clones were rescued per virus. (B) Western blot detection confirms expression of the SARS-CoV-2 spike S1 domain at 110 kDa and GFP at 26.9 kDa, and an image of the cell pellets at P3 further confirms GFP is expressed. (C) Side-by-side growth kinetics of BAC6-polh(S1), FBG-polh(S1), and BAC6-e56(GFP)-polh(S1). Three biological replicates were included during the assay. Samples were taken at 0, 24, 48, 72, 96, and 120 hpi and titrated on Sf9-ET cells. The data show the mean ± SEM of triplicates. (D-E) Suspension-cultured Sf9 cells were infected with recombinant virus at an MOI of 5. At 72 hpi the culture fluid was harvested, and the S1 domain was quantified with ELISA. The productions were performed in experimental duplicate, with three or four biological duplicate clones per virus. The data shows the mean ± SEM.

Having characterized BAC6, we next sought to investigate the utilization of the bacmid for protein expression. SARS-CoV-2 spike S1 domain productions were performed with BAC6 and *FBG* to compare to a commercial standard. A transgene consisting of a *polh* promoter-driven coding sequence of the SARS-CoV-2 spike S1 domain^9^ was introduced into BAC6 and FBG through homologous recombination at the polh locus. Additionally, a second BAC6-based virus was included, in which a second transgene (eGFP) was introduced at the odv-e56 locus through transposition (Figure 6A). Per virus, three or four plaque purified clones were amplified to P3 and transgene expression was confirmed with a western blot of the virus stocks to be used for production (Figure 6B). The SARS-CoV-2 S1 domain has an expected mass of 110 kDa, which is observed for all clones. The eGFP transgene is only expected in the BAC6-e56(GFP)-polh(S1) clones, at a mass of 26.9 kDa. The expression of GFP was detected on a western blot, as well as visually in the cell pellets of the P3 stocks.

To confirm whether the transgene presence affects the growth kinetics of the viruses, growth curves were generated in the same manner as described before. The generated growth curves revealed comparable growth kinetics for the viruses in the study (Figure 6C). No significant differences were observed between the replicates or the different viruses at any time point. When comparing the growth kinetics of the viruses in this study to those of the viruses in Figure 1E, at 48 hpi the titers of the viruses in the S1 production study are lower compared to the viruses in the earlier study. Since the lower titer at 48 hpi is observed in all viruses used in the S1 production experiment and the titers at the other time points were more in line with expectations, this was not further analyzed, and protein production was continued.

To produce the S1 spike protein, suspension cultures of Sf9 cells were infected with the recombinant viruses. The S1 protein concentrations were determined with ELISA (Figures 6D and 6E). For BAC6-polh(S1) and FBG-polh(S1) (Figure 6D) comparable amounts of S1 were detected in the medium, averaging at 6.22 and 6.99 μg/mL, respectively. Slightly higher S1 titers were detected in the culture fluid of productions with BAC6-e56(GFP)-polh(S1) (Figure 6E), on average 10.6 μg/mL. The spike S1 domain productions demonstrated that the BAC6 has comparable productivities to a commercial standard and that co-expression with a second *polh* promoter-driven transgene from the odv-e56 locus does not negatively influence transgene production.

### Recombinant AAV production by BAC6

**Figure 7:**
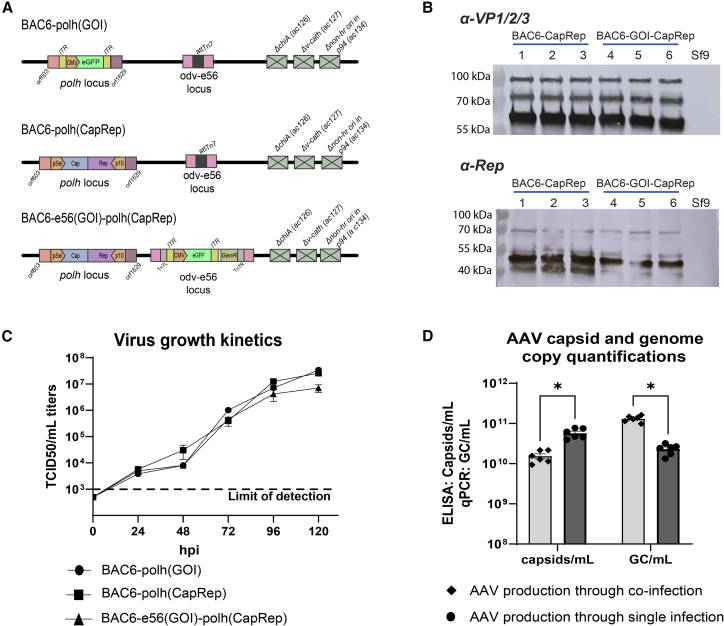
rAAV production results with the novel bacmid (A) Three viruses were included in the study. BAC6-polh(GOI) and BAC6-polh(CapRep) both contain a transgene only at the polyhedrin locus and can produce AAV when co-infecting the two stocks together. BAC6-e56(GOI)-polh(CapRep) contains all the necessary components for AAV production in one virus. Three plaque-purified clones were rescued per virus. (B) Western blot expression of VP1, VP2, and VP3 is expected at 87, 72, and 62 kDa, respectively, in a 1 (VP1):1 (VP2): 10 (VP3) ratio. Rep78 and 52 are expected at 70.5 and 44.7 kDa, respectively. (C) Virus growth kinetics were investigated for all viruses in the study with EPDA. The data show the mean ± SEM of triplicates. (D) Suspension-cultured Sf9 cells were infected with one or two recombinant virus(es) at an MOI of 5 (co-infection) or 10 (single infection). At 72 hpi, the cells were lysed and the supernatant was sampled. Genome copy titers were determined with qPCR (genome copies/mL) and AAV capsid titers with ELISA (capsids/mL). The productions were performed in duplicate, with three clones per virus. Significance was determined by unpaired t-tests in Graphpad Prism Version 10.4.2. ∗: *p* < 0.05. The data show the mean ± SEM.

To complement the S1 production test, a second study was performed through the production of recombinant AAVs. BAC6 could be attractive for this purpose due to the availability of two transgene insertion sites, allowing for flexibility for the introduction of the AAV cap, rep, and inverted terminal repeat (ITR)-flanked gene of interest (GOI) sequences. Three viruses were rescued, which contain one or two AAV transgenes (BAC6-polh(CapRep) and BAC6-polh(GOI)) or all three AAV transgenes (BAC6-e56(GOI)-polh(CapRep)) in a single baculovirus (Figure 7A). In both BAC6-polh(CapRep) and BAC6-polh(GOI), the transgenes were introduced at the *polh* locus through homologous recombination. In BAC6-e56(GOI)-polh(CapRep), the GOI is introduced at the odv-e56 locus through transposition and the CapRep cassette through homologous recombination at the *polh* locus. Per virus, three plaque-purified clones were amplified to P3, and transgene expression was confirmed on western blots (Figure 7B). AAV VP1, VP2, and VP3 were detected upon infection with BAC6-polh(CapRep) and BAC6-e56(GOI)-polh(CapRep) at sizes of 87, 72, and 62 kDa, respectively. Compared to VP1 and VP2, there is more VP3 produced, which is in line with expectations (Urabe et al., 2002). The major replication protein Rep78 and the minor replication protein Rep52 are both detected on a western blot at the expected 70.5 and 44.7 kDa, respectively. An additional band slightly below Rep52 is also observed, which could possibly be Rep40. Growth curves were generated in triplicate by infection of adherent layers of Sf9 cells at an MOI of 0.5. Time point samples were taken from the supernatant from 0 until 120 hpi at 24 h intervals and titrated on Sf9-ET cells. No significant differences were observed among the replicates or between the different viruses (Figure 7C). At the final 120 hpi time point, the EPDA titer of BAC6-e56(GOI)-polh(CapRep) appeared lower compared to BAC6-polh(CapRep) and BAC6-polh(GOI), but this difference was not significant. Similar to the S1-producing viruses, the lower titers at the 48 hpi time point were noted again. The EPDA titer determinations were carried out for the S1 and rAAV-producing viruses in the same study. Similarly, since the lower titers were only observed at 48 hpi and restored after, production was continued.

For rAAV production, suspension cultures of Sf9 cells at a density of 1e6 cells/mL were co-infected with BAC6-polh(CapRep) and BAC6-polh(GOI), both with an MOI of 5, or infected with BAC6-e56(GOI)-polh(CapRep) at an MOI of 10. At 72 hpi, the cells were lysed, the cell debris was pelleted by centrifugation, and the supernatant was sampled. The rAAV production experiments were performed twice in triplicate per condition (single or co-infection). The capsid titers were determined in capsids/mL with ELISA and packaged GOI genomes in genome copies (GC)/mL with qPCR (Figure 7D). The rAAV production through co-infection yielded average capsid titers of 1.54e10 capsids/mL and genome copy titers of 1.32e11 GC/mL. For the single infection production with BAC6-e56(GOI)-polh(CapRep), average capsid titers of 5.8e10 capsids/mL and genome copy titers of 2.3e10 GC/mL were found. The capsid/mL titers of the single infection approach are significantly higher compared to the co-infection approach, although for the GC/mL titers this was the opposite. In the case of the co-infection approach, the GC/mL titer, which quantifies encapsidated genomes, is higher compared to the capsids/mL titer. Since the titers for capsid and GC were determined through separate approaches (ELISA and qPCR, respectively), they cannot be used to accurately calculate empty/full ratios, although separately they can be studied. Together, these productions show that BAC6 is capable of producing rAAVs through both co-infection and single-infection strategies, although optimization of the production process conditions is required to improve the yields to industrial standards.

## Discussion

This study aimed to develop a novel, genetically stable baculovirus vector (bacmid) that is compatible with the incorporation of transgenes at two different loci. Using lambda red recombineering in *E. coli*, the attTn7 transposition site was relocated to the odv-e56 locus, and partial deletions in the ORFs flanking the *polh* locus were introduced to improve compatibility with homologous recombination in insect cells. The use of homologous recombination at the *polh* locus is well described and has since been implemented in various classical bacmid systems.^18^ The benefit of this homologous recombination step is that the mini-F replicon, an ∼8 kb sequence required for bacmid replication in *E. coli* but dispensable for bacmid-derived baculovirus replication in insect cells, is absent from the recombinant baculovirus genomes. The mini-F region was previously shown to be a major determinant of transgene instability,^34^ and relocation of the attTn7 site from the mini-F to another locus, including odv-e56, was demonstrated to be beneficial for protein expression.^35^ To improve transgene secretion and prevent accidental product degradation, v-cath and chiA ORFs were also deleted. Lastly, to improve the genetic stability and delay DIP formation, the non-hr ori in p94 was removed. The resulting bacmid, BAC6, performed similarly compared to a commercial standard (FBG; Oxford Expression Technologies) in a high MOI genetic stability study and has comparable growth kinetics. BAC6 also produced comparable levels of SARS-CoV-2 spike S1 domain compared to a commercial control based on homologous recombination only. Lastly, the production of high rAAV yields was demonstrated through co-infection with two baculovirus vectors, as well as through infection of Sf9 cells with a single bacmid carrying all necessary functions.

Passaging is inevitable in order to increase the titer and volume of virus stocks, which is required for larger-scale recombinant protein production. Genetic stability of the baculovirus is important during the passaging of virus stocks to ensure final product yield and quality. A minimum number of five to seven passages are generally required in order to scale up the virus for large-scale commercial manufacturing; therefore, the genetic stability that we were aiming for in this study was at least P8. Instability can, for example, be caused by a lack of selection or a negative selection pressure on transgene sequences, as well as instability of the viral genome itself, resulting in the generation of deletion mutants and DIP formation. Serial undiluted passaging at high MOI was chosen to study the virus dynamics, promote the formation and accumulation of DIPs, and test whether the deletion of the non-hr ori in p94 would be sufficient in delaying DIP formation for 8–10 passages.^26^ During the passaging experiment described in this study, the negative effects of DIP formation on transgene expression and virus infectivity were observed in one out of two clones of BAC6-mCherry from P7/P8 onwards. This was observed through a gradual reduction in fluorescence, and cytopathic effects (CPE) was observed under the microscope, with nearly none left at P10. Other causes were ruled out by the results of EPDA, GP64 mAb titration, and low MOI infection experiments. Clone 1 in the EPDA also shows titers that are decreasing from P7 onward, which is an expected observation when dealing with a stock contaminated with DIPs.^27^

Despite only observing the effects of DIP formation from P7/P8 onwards in the microscopy and titration datasets, the NGS on viral DNA shows the accumulation of a DIP population to already be present at P3 in clone 1. The plots from P3 to P10 show increasing enrichment of read coverage over the genome from about ∼60.000 nt onwards, meaning an increase over approximately 56% of the genome (∼60.000 until 137.108 nt) and decrease over the remaining 44% of the genome (0 until ∼60.000 nt). Kool et al. described a similar deletion mutant that misses approximately 43% of the wild-type AcMNPV genome (strain E2) that arose during a continuous production experiment.^40^ Earlier, Carstens and Brown et al. also described a defective AcMNPV mutant with a similar deletion of approximately 42% in the same region as the formerly mentioned.^41^^,^^42^ The results of the study highlight the value of screening early passages of promising clones with NGS. Illumina NGS has the benefits of a high coverage depth, low error rate, and lower sample input requirements; however, for assembling the genomes of (defective) deletion mutants within viral stocks that also contain viruses with full-length genomes, a long-read sequencing technique, such as nanopore or PacBio sequencing, might be more suitable, as that is less affected by the baculovirus hr-domains.

In BAC6 the non-hr ori in p94 was deleted to prevent enrichment of DIPs in this region and hopefully delay DIP formation based on earlier findings.^14^ In BAC6-derived viruses, this mutation indeed prevented this specific DIP population from forming, although a different DIP (lacking 44% of the genome) took its place instead, as discussed above. Therefore, the observed decrease in mCherry expression under the microscope in BAC6-mCherry clone 1 is likely caused by the enrichment of DIPs within the stock that also no longer contain the transgene sequence. In the commercial control that was taken along in the passaging experiment (FBG-mCherry), the non-hr ori in p94 was not deleted. NGS analysis of the FBG-mCherry clones revealed an increase in coverage over a region in the genome spanning from approximately 108.000 to 119.500 nt. Within this region are the ORFs *ac130* through *ac139*. Since the non-hr ori within the p94 gene (*ac134*) falls within this region, it is likely that for these clones the DIP population originated there. The NGS data highlighted that removal of the non-hr ori from p94 is an effective way to prevent at least this specific DIP population from forming.

Furthermore, in the FBG-mCherry virus passages, additional populations were observed. In the first clone, a deletion in the genome from ∼150 to 3000 nt was detected, encompassing part of *hr 1* until halfway through *ac5*, which likely does not result in a defective virus population, since *hr 1* as well as the region from *ac1 to ac5* are all non-essential for replication in cell culture and BV generation.^43^^,^^44^^,^^45^^,^^46^^,^^47^^,^^48^ In clone 2 a deletion from ∼3000 to 4700 nt is observed. This covers part of *ac5* until the first 130 bp of the mCherry transgene sequence placed at the *polh* locus (*ac8*), which explains why there was a difference in GFP and mCherry readouts in the EPDA assay. In the NGS data, this deletion is already observed in P6 and found more prominently in P10. Besides part of the transgene, the *lef-2* (*ac6*) gene is also within the deleted region. LEF-2 is essential for virus replication, and deletion is reported to be lethal for the virus.^39^ It is likely that due to the *lef-2* deletion, this genotype represents a DIP population that is co-replicating along with the intact virus. Genetic stability for FBG and BAC6 viruses was tested with only two clones per virus. More clones should be tested to conclude whether the observed populations are chance events or that the bacmid induces statistically more or less DIP formation.

There is room for further optimization in terms of genetic stability during extensive passaging under high MOI conditions, although typically passages to scale up the virus stock are carried out at low MOI, where the amount of DIPs expected to be observed is minimal. Further research into the deletion of additional ORFs associated with genomic instability, such as fp25k^30^ and da26^20^ is worthwhile. Additionally, decreasing the genome size even further by removal of regions that are non-essential for BV generation might also improve genomic stability.^31^^,^^32^ This potentially also increases available space to allow for the introduction of more/larger transgene inserts.

To test the production competences of BAC6, SARS-CoV-2 spike S1 domain and rAAV production experiments were performed. For the spike S1 domain, comparable culture fluid S1 yields of ∼6 μg/mL were obtained with BAC6 and FBG, thereby proving that the novel bacmid has comparable production capacities to the commercial control. With the BAC6 virus co-expressing GFP and S1, a slightly higher yield of ∼11 μg/mL. This virus was included to demonstrate that co-expression does not negatively impact S1 production. The slightly increased S1 production in the ELISA was unexpected but might result from better secretion and/or increased cell death compared to the other viruses in the experiment. An S1 yield of 11 μg/mL was also obtained in the production described by Van Oosten et al. using the FBG vector.^9^ Although the protocol differed from this study, the yields are similar to the current study.

The rAAV production with BAC6 showcased effective production through co-infection as well as single infection. Significantly higher capsid titers (>3-fold) were reached with the single infection approach, compared to co-infection. On the contrary, the GC/mL titers obtained in the co-infection were significantly higher (>5-fold) compared to the single infection. The GC/mL titers (determined by qPCR) achieved with the co-infection approach were higher than the recorded capsid/mL titers (determined by ELISA). Alternative quantification methods, such as mass photometry or analytical ultracentrifugation, may be used to determine more accurate AAV empty:full ratios.

Production of rAAVs with a single baculovirus has been described before in literature. Wu et al. generated a recombinant baculovirus by introducing Cap, Rep, and GOI into the *polh* locus of a bacmid through transposition.^49^ Galibert et al. transposed an ITR-human gamma-sarcoglycan transgene at the *polh* locus and introduced Cap and Rep into the egt locus (*ac15*) through lambda red recombineering in *E. coli*.^50^ Both of these studies use similar approaches of transgene introduction as the current study. Comparison between the presented bacmid and the two literature examples is difficult due to different experimental setups, the absence of capsid quantification in Wu et al., and Galibert et al. only reporting capsid and genome copy titers after purification and concentration. In general, the approach of producing rAAVs through a single infection seems promising, as it allows low-MOI infection strategies in large-scale bioreactors, although it requires some optimization and additional investigation into the specifics of interaction between the baculovirus and AAV transgenes.

The significantly higher genome copy titers in the co-infection experiment might suggest that the replication of the ITR-flanked transgene might be more efficient when present in a separate baculovirus genome. When Rep78 facilitates ITR-GOI replication, it nicks in the ITR, which would cause excision of the transgene sequence from the baculovirus backbone.^51^ This probably affects the replication of the all-in-one baculovirus vector and, as a consequence, leads to reduced gene expression. Potentially this can be solved by placing Rep78 under an inducible promoter to allow for more control in gene expression. In addition, the MOIs varied between the different conditions, since for the co-infections an MOI of 5 was used for both viruses, and for the single virus an MOI of 10 was used. A benefit of using a co-infection system for rAAV production is that it allows for more flexibility in experimental conditions, such as optimizing the ratio between the two viral vectors by individually adapting the MOIs, which is not possible with a single baculovirus system. Overall, the proof-of-concept rAAV productions demonstrated that BAC6 can produce high titers of rAAVs, both in a co-infection as well as in a single-infection setup, and its applications go beyond.

## Materials and methods

### Baculovirus genome editing

The lambda red engineering technique as described in De Jong et al.^13^ was applied to generate the mutations described in Table 1 into the AcMNPV bacmid bMON14272.^10^ Briefly, bMON14272 bacmid DNA was electroporated into *E. coli* harboring a λ Red operon (GB05-Red^52^), generating GB05-Red-BAC. An rpsL-CmR counter-selection cassette with homology to the target region was amplified with PCR and electroporated into competent GB05-Red-BAC cells. Bacteria containing the recombinant bacmids were screened with colony PCR after positive selection on LB agar supplemented with 20 μg/mL chloramphenicol. In a similar manner, the selection cassette was removed by electroporating “marker removal DNA” consisting of sequences with homology to the regions flanking the cassette in the bacmid. Colonies were screened with colony PCR after negative selection (for absence of the rpsL gene) on 100 μg/mL streptomycin to confirm scarless removal of the cassette. The mutations were introduced sequentially to generate BAC6. The design of the ORFs flanking the *polh* locus is based on Kitts et al.,^18^ the deletion of v-cath and chi-A on Kaba et al.,^33^ the deletion of the non-hr ori in p94 on Pijlman et al.,^14^ and the relocation of the attTn7 site on Pijlman et al.^35^ The resulting BAC6 bacmid DNA was isolated from *E. coli* and analyzed using NGS with the Illumina NovaSeq X platform for 150-bp paired-end sequencing at Macrogen Europe. Illumina NGS libraries have been uploaded to the NCBI sequence read archive (SRA) under BioProject ID: PRJNA1406022. The raw data was clipped with fastp^53^ to remove adapters and low-quality reads (threshold quality value = 20; read length threshold = 25). The clipped dataset was mapped with the Segemehl^54^ mapper using default settings to wild-type AcMNPV strain E2 (GenBank accession number KM667940.1) and the sequence of the mini-F replicon to generate a reference sequence for later experiments.

For transposition, the bacmid DNA was electroporated into DH10β cells harboring the pMON7124 transposase helper plasmid.^10^ The cells were made competent and electroporated with a donor plasmid to introduce the first transgene into the atTTn7 site at the odv-e56 locus. Positive selection was done on 7 μg/mL gentamicin, and colonies were screened with colony PCR. Correct colonies were inoculated in LB medium supplemented with kanamycin (50 μg/mL) and gentamicin (7 μg/mL) and incubated in a 37°C shaking incubator overnight. DNA was isolated the following day with standard alkaline lysis. The introduced GOI was either a *polh* promoter-driven *eGFP* gene or a CMV promoter-driven eGFP sequence flanked by AAV serotype 2 ITRs, depending on the application described further below.

### Insect cell culture

*Spodoptera frugiperda* 9 (Sf9) cells were grown in EX-CELL chemically defined insect cell medium (Sigma-Aldrich). Sf9 easy titer (Sf9-ET^55^) cells (Kerafast) were grown in Sf900II serum-free medium (Gibco) supplemented with 50 μg/mL geneticin (Gibco). Both cell lines were cultured in suspension in 125 mL shake flasks in a working volume of 30 mL in an incubator at 27°C on an orbital shaker platform set at 110 rpm. The cells were subcultured twice per week when the concentrations exceeded 4e6 cells/mL to maintain the cells in an exponential growth phase.

### Virus rescue and propagation

Bacmid DNA was co-transfected with a transfer plasmid using the ExpresS2 TR Transfection Reagent (Expression Systems). Briefly, a mix of 95 μL EX-CELL medium and 5 μL transfection reagent was prepared in an Eppendorf tube. A second mix contained 200 ng bacmid and 500 ng transfer plasmid DNA in a total volume of 100 μL EX-CELL medium. The transfection reagent mix was added to the DNA mix, and the resulting mixture was incubated at room temperature for 15 min before being used to transfect adherent Sf9 cells seeded in a 6-well plate at a density of 1.5e6 cells/well. At 5 days post transfection (dpt), the supernatant was harvested (passage (P) 0) and used in a plaque assay to obtain homogenized virus stocks. In short, Sf9 cells were seeded in 6-well plates at a density of 1.5e6 cells/well and subsequently infected with a dilution range of the transfection harvests. One hpi, the inoculum was replaced with 2 mL of a pre-warmed 1:1 mix of 2% UltraPure Low Melting Point Agarose (Invitrogen) and double-concentrated EX-CELL medium. The agarose layer was left to solidify at room temperature for 15 min. The plates were transferred to a 27°C static incubator and incubated for 5–7 days. Single plaques were picked with a sterile pipet tip and resuspended in 500 μL EX-CELL medium (P1). Plaques were amplified by using 250 μL of the resuspended plaques to infect new Sf9 cells in 6-well plates seeded at a density of 1e6 cells/well. Amplifications were harvested 3–5 dpi (P2) depending on the progression of observed CPE. Virus titers in tissue culture infectious dose per mL (TCID50/mL) were determined by EPDA on Sf9-ET cells.^55^

### Baculovirus growth kinetics

To analyze the growth kinetics, an adherent monolayer of Sf9 cells seeded in a 6-well plate at a density of 5e5 cells/well was infected in triplicate with an MOI of 0.5. After incubation at 27°C on a tilting platform for one hour, the virus inoculum was replaced with culture medium. Supernatant of the infected cell cultures was sampled at time points 0, 24, 48, 72, 96, and 120 hpi and stored at −20°C. Baculovirus titers were determined by EPDA on Sf9-ET cells.

### mCherry quantification with flow cytometry

Two recombinant viruses were included in this experiment, generated with either the novel BAC6 backbone or with the FBG (Oxford Expression Technologies) bacmid. The viruses contain an mCherry transgene under the *polh* promoter, introduced via homologous recombination during co-transfection on insect cells. Per virus, three plaque-purified clones were amplified to P3, titrated with EPDA, and used to infect 1e6 cells/mL suspension cultures of Sf9 cells with an MOI of 5. At 24, 48, and 72 hpi, 1 mL samples were collected from each flask and used for measuring the shift in mean fluorescence intensity (MFI) caused by mCherry transgene expression with the CytoFlex Flow Cytometer (Beckman Coulter) equipped with a B610_20-A filter for mCherry detection. A culture of uninfected Sf9 cells was sampled along as a negative control. The data was analyzed with the CytExpert software (v2.5.0.77; Beckman Coulter) and plotted with GraphPad Prism (v10.4.2).

### Genetic stability high MOI passaging experiment

Two clones of the BAC6-mCherry and FBG-mCherry virus stocks previously used for the flow cytometer experiment were serially passaged at high MOI to investigate genetic stability. Monolayers of Sf9 cells were seeded in T25 flasks at a density of 1.8e6 cells/flask in 3 mL EX-CELL medium. The P3 stocks were used to generate P4 at an MOI of 10. At 3 dpi, 1 mL of undiluted supernatant from the flask was used to infect the next passage until P10 was reached. The supernatant harvest of each passage was aliquoted and stored at −80°C, later to be used for titer determination with endpoint dilution assays on SF9ET cells (as described above), and through a flow cytometric approach after staining with the Baculovirus Envelope GP64 Monoclonal Antibody (AcV1) (eBioscience). At each passage, cells were monitored for CPE and mCherry transgene expression prior to harvest. Additionally, the cell pellets from each of the passages were collected and stored in phosphate-buffered saline (PBS) buffer at −20°C to be used for DNA isolation with the DNeasy Blood & Tissue Kit (Qiagen). Isolated viral DNA samples from passages 3, 6, and 10 were sent for NGS with the same specifications described for the bacmid before. Illumina NGS libraries have been uploaded to the NCBI sequence read archive (SRA) under BioProject ID: PRJNA1406022. The raw data was clipped with fastp^53^ to remove adapters and low-quality reads (threshold quality value = 20; read length threshold = 25). The clipped dataset was mapped to the reference sequence with the Segemehl mapper^54^ using default settings. The coverage over each nucleotide was normalized between the datasets to account for differences in sequencing depth, and the coverages were plotted with gnuplot.^56^

### Recombinant SARS-CoV-2 S1-domain protein productions

An eGFP driven by a *polh* promoter was transposed into the attTn7 site in the novel bacmid to generate BAC6-e56(eGFP). Bacmid DNA was purified from *E. coli* using a standard alkaline lysis protocol. Insect cells were then co-transfected with BAC6-e56(GFP), BAC6, or FBG together with a transfer plasmid compatible for homologous recombination at the *polh* locus. The transfer plasmid contains the SARS-CoV-2 S1 spike domain (Sp53).^9^ Virus stocks were plaque purified and screened with PCR and western blot analysis. Next, the stocks were amplified, titrated, and subsequently used to infect (MOI 5) suspension cultures of Sf9 cells, seeded at a density of 1e6 cells/mL in a total volume of 25 mL. The infections were incubated at 27°C on an orbital shaker platform set at 110 rpm. At 3 dpi, the shake flasks were harvested by transferring the contents to 50 mL Falcon tubes and centrifuging at 1000×*g* for 5 min to pellet cells and cell debris. Freezing solution (50% sucrose (w/v) and 1% pluronic (v/v) in MilliQ water; sterilized through a 0.2 μM filter) was added in a 1:10 (freezing solution: sample harvest) ratio, and the samples were stored in aliquots at −80°C. The cell pellets were resuspended in 1 mL PBS buffer and stored at −20°C. The cell pellets were used to prepare protein samples for sodium dodecyl sulfate polyacrylamide gel electrophoresis (SDS-PAGE) under denaturing conditions by addition of dithiothreitol (DTT) to a final concentration of 50 mM/mL. Samples were boiled at 95°C for 10 min before being loaded on Any kD Mini-PROTEAN TGX Stain-Free Protein Gels (Bio-Rad). Proteins were blotted on a nitrocellulose membrane (Invitrogen). The membranes were blocked in 1% skim milk powder (Campina) in 0.1% Tween 20 in PBS (PBST) buffer. SARS-CoV-2 (2019-nCoV) Spike Antibody (Sino Biological; cat#40591-T62), the primary antibody for spike detection, was added in a 1:5000 dilution in 1% skim milk powder in PBST. As a secondary antibody, alkaline phosphatase-labeled goat anti-rabbit IgG secondary antibody (Invitrogen; cat#656122) was added in a 1:10000 dilution in PBST. Detection of the alkaline phosphatase-labeled proteins was done through NBT-BCIP staining (Roche Diagnostics GmbH). The produced spikes were quantified with the SARS-COV-2 Spike S1 Protein ELISA Kit (AssayGenie; cat# CBK4154). Data was plotted using GraphPad Prism.

### Recombinant AAV production

A CMV promoter-driven eGFP flanked by AAV2 ITRs (hereafter referred to as GOI) was transposed into the attTn7 site in the novel bacmid to generate BAC6-e56(GOI). Bacmid DNA was purified from *E. coli* using a standard alkaline lysis protocol. An empty BAC6 bacmid and the BAC6-e56(GOI) were each co-transfected with a transfer plasmid to introduce AAV Cap and Rep at the *polh* locus, yielding BAC6-polh(CapRep) and BAC6-e56(GOI)-polh(CapRep). Lastly, a BAC6 bacmid was co-transfected with a transfer plasmid to introduce GOI at the *polh* locus to generate BAC6-polh(GOI). The transfer plasmid used for CapRep introduction into the baculovirus contains a p10 promoter driving AAV2 replication (Rep) proteins 78 and 52,^57^ and the VP1/VP2/VP3 viral capsid (Cap) protein coding sequence driven by a pSel promoter.^58^ Similar as described in Dudek et al., chimeric AAV2.5 capsids were produced in which the VP1 unique region from AAV5 was swapped with one from AAV2.^59^ Virus stocks were plaque purified and screened for transgene presence with PCR, and expression was verified with western blot analysis with primary antibodies against AAV2 Rep (Progen; Cat#61073) and AAV2 VP1/2/3 (Progen; Cat#690058S). Alkaline phosphatase-labeled anti-Mouse IgG antibody (Sigma-Adrich; cat#A5153) was used as a secondary antibody. The stocks were amplified at low MOI to P3, titrated with EPDA, and subsequently used for rAAV production in suspension cultures of Sf9 cells, seeded at a density of 1e6 cells/mL in a total volume of 25 mL. For the co-infection approach, both BAC6-polh(CapRep) and BAC6-polh(GOI) were infected at an MOI of 5 (total MOI 10). For the single infection approach, BAC6-e56(GOI)-polh(CapRep) was infected at an MOI of 10. The infections were incubated at 27°C on an orbital shaker platform set at 110 rpm. At 3 dpi, the shake flasks were harvested by addition of 1% (v/v) lysis buffer (250 mM MgCl2, 0.05% Pluronic F68, and 1% Triton X-100 in 2M Tris buffer (pH 8)), followed by a 1-h incubation step at 27°C and 110 rpm before pelleting the lysate by centrifugation at 3000 ×g for 30 min. The supernatant was stored in aliquots at −80°C. The produced AAV capsids were quantified with an AAV5 Titration ELISA (Progen; Cat#PRAAV5), and GC was quantified with the qPCR AAV Titer Kit (ABM; Cat#G931). Data was plotted using GraphPad Prism.

## Data and code availability

The authors confirm that data supporting the findings and conclusions of this study are presented within the article.

## Acknowledgments

This work was funded by VectorY Therapeutics. We thank Hilde van Tongeren for her input during the early stages of the projects. We also thank Astrid Coolen, Magdalena Nazaruk, Elisa Teunissen, and Michelle van Meerendonk for technical assistance during analyses performed at VectorY Therapeutics.

## Author contributions

L.A.d.J. conceived and performed experiments, analyzed the data, and wrote and edited the manuscript. M.S., J.W., A.H.d.W., D.E.M., and B.P.S. contributed to the conception and design of experiments, and edited the manuscript. C.G. and M.H. contributed to performing experiments. M.M.v.O. acquired the funding and edited the manuscript. G.P.P. contributed to the conception and design of experiments, supervised the project, and edited the manuscript.

## Declaration of interests

J.W., A.H.d.W., and B.P.S. are employees of VectorY Therapeutics. The other authors declare no competing interests.
